# Genomic aberrations in borderline ovarian tumors

**DOI:** 10.1186/1479-5876-8-21

**Published:** 2010-02-26

**Authors:** Francesca Micci, Lisbeth Haugom, Terje Ahlquist, Hege K Andersen, Vera M Abeler, Ben Davidson, Claes G Trope, Ragnhild A Lothe, Sverre Heim

**Affiliations:** 1 Section for Cancer Cytogenetics, Institute for Medical Informatics, The Norwegian Radium Hospital, Oslo University Hospital, Oslo, Norway; 2 Department of Cancer Prevention, Institute for Cancer Research, The Norwegian Radium Hospital, Oslo University Hospital, Oslo, Norway; 3 Centre for Cancer Biomedicine, University of Oslo, Oslo, Norway; 4 Department of Pathology, The Norwegian Radium Hospital, Oslo University Hospital, Oslo, Norway; 5 Department of Gynecology, The Norwegian Radium Hospital, Oslo University Hospital, Oslo, Norway; 6 Faculty of Medicine, University of Oslo, Oslo, Norway

## Abstract

**Background:**

According to the scientific literature, less than 30 borderline ovarian tumors have been karyotyped and less than 100 analyzed for genomic imbalances by CGH.

**Methods:**

We report a series of borderline ovarian tumors (n = 23) analyzed by G-banding and karyotyping as well as high resolution CGH; in addition, the tumors were analyzed for microsatellite stability status and by FISH for possible 6q deletion.

**Results:**

All informative tumors were microsatellite stable and none had a deletion in 6q27. All cases with an abnormal karyotype had simple chromosomal aberrations with +7 and +12 as the most common. In three tumors with single structural rearrangements, a common breakpoint in 3q13 was detected. The major copy number changes detected in the borderline tumors were gains from chromosome arms 2q, 6q, 8q, 9p, and 13q and losses from 1p, 12q, 14q, 15q, 16p, 17p, 17q, 19p, 19q, and 22q. The series included five pairs of bilateral tumors and, in two of these pairs, informative data were obtained as to their clonal relationship. In both pairs, similarities were found between the tumors from the right and left side, strongly indicating that bilaterality had occurred via a metastatic process. The bilateral tumors as a group showed more aberrations than did the unilateral ones, consistent with the view that bilaterality is a sign of more advanced disease.

**Conclusion:**

Because some of the imbalances found in borderline ovarian tumors seem to be similar to imbalances already known from the more extensively studied overt ovarian carcinomas, we speculate that the subset of borderline tumors with detectable imbalances or karyotypic aberrations may contain a smaller subset of tumors with a tendency to develop a more malignant phenotype. The group of borderline tumors with no imbalances would, in this line of thinking, have less or no propensity for clonal evolution and development to full-blown carcinomas.

## Introduction

Borderline ovarian tumors are of low malignant potential. They exhibit more atypical epithelial proliferation than is seen in adenomas, their benign counterpart, but are without the destructive stromal invasion characteristic of overt adenocarcinomas [1]. Although the clinical and pathological features of tumors of borderline malignancy thus are intermediate, it is not clear whether they represent a transitional form between adenomas and invasive carcinomas, as a stage in multistep carcinogenesis, or alternatively, whether all three tumor types should be regarded as independent entities brought about by different molecular mechanisms [1,2].

Although a comparison of the cytogenetic abnormalities occurring in ovarian carcinomas and tumors of borderline malignancy could provide insights into their pathogenetic relationship, little information is available on the karyotypic patterns of the latter tumors. Indeed, whereas chromosomal abnormalities have been reported in over 400 ovarian carcinomas [3], the corresponding cytogenetic information on borderline tumors is limited to only 27 cases [4-11]. Karyotypic simplicity with few or no structural rearrangements seems to be characteristic with trisomies for chromosomes 7 and 12 as the most common abnormalities [6-9]. Using fluorescent in situ hybridization (FISH), Tibiletti et al. [2] found consistent loss of a small area of 6q in a high percentage of borderline ovarian tumors.

Several studies have used comparative genomic hybridization (CGH) to identify the imbalances present in tumor genomes, also in the ovarian context. Of nearly 100 borderline tumors analyzed, half have shown genomic imbalances. The most frequent abnormalities thus detected have been gains of or from chromosomes 5, 8, and 12 and losses from 1p [12-17].

We here report a series (n = 23) of borderline ovarian tumors analyzed by G-banding, high resolution (HR)-CGH, FISH-examination for possible 6q deletions and 3q rearrangements, and a microsatellite instability (MSI) assay. The latter analysis was included because ovarian cancer can be part of the hereditary non-polyposis colon cancer (HNPCC) spectrum which is often characterized by MSI.

## Materials and methods

### Tumors

**Figure 1:**
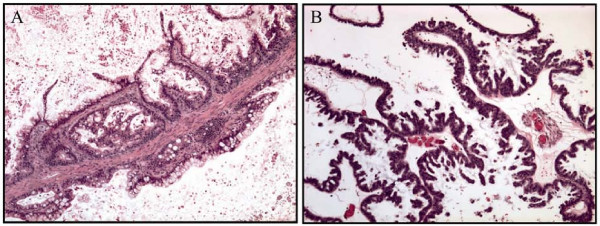
**Histological sections from case 17 (a) a mucinous and case 7 (b), a serous borderline ovarian tumor**.

The examined material consisted of 23 fresh samples from ovarian tumors surgically removed at The Norwegian Radium Hospital from 2001 to 2004 (Table 1). The tumors were all classified as borderline, either with serous (17 cases, Fig. 1), mucinous (5 cases), or a mixed serous and mucinous differentiation (case 18). In five patients, bilateral borderline tumors were analyzed (cases 7 and 8, 9 and 10, 13 and 14, 19 and 20, and 22 and 23; hence, the total number of patients was 18). The utilization of the tumor material for research purposes was approved by institutional as well as regional ethical committees.

**Table 1:** Borderline Ovarian Tumors Examined by Karyotyping, High Resolution-CGH, and Microsatellite Instability Analysis.

Case num/lab num	Type	Surface	Extraovarian	Karyotype	Genomic imbalances	MS status
1/01-642	mucinous	no	no	47, XX, +12[4]/47, XX, +7[3]/45, XX, -6[3]/46, XX[63]	rev ish enh(1q22q32, 2p25, 2q22q24, 2q32q33, 3p12p14, 3p22, 3p23, 3p24, 3q12q13, 3q24, 3q25, 5p14, 5q14q22, 6q12q21, 6q22q23, 8q13, 8q21, 8q22q24, 9p13p21, 9p23, 10q21, 18q12), dim(1p21, 1p31pter, 7q11, 11p15, 11q12q14, 11q23, 12q23, 12q24, 13q12, 13q14, 13q33q34, 14q21q24, 14q31q32, 15q13q14, 15q22q24, 17p11p13, 17q, 19p13, 19q, 22q11q13)	MSS

2/01-700	mucinous	no	no	46, XX[116]	rev ish enh(8q23, 9p23), dim(1p34p35, 7q11, 17p12p13, 19p13, 19q13, 22q11q12)	MSS

3/01-839	serous	yes	non-invasive implants	46, XX, t(3;17) (q13;q24)[2]/46, XX[45]	rev ish enh(3p13, 9p23p24), dim(1p33pter, 7q11, 9q34, 11q13, 12q24, 16p11p13, 17p12pter, 17q12q21, 19p13, 19q, 22q11q13)	no DNA available

4/01-844	mucinous	no	no	46, XX, +12, -22[7]/46, XX[19]	no DNA available	MSS

5/02-1	serous	yes	no	46, XX[16]/92, XXXX[23]	rev ish enh(2q22q24, 2q31q32, 3p12, 3q12q13, 4p15, 4q13, 5p14, 5q14q23, 6q15q16, 8q22, 8q23, 13q21q31, 13q32, 21q21), dim(1p32pter, 2q37, 3p21, 4q35, 5q35, 6p21, 6p22, 6q25, 7q11, 9q22, 9q33qter, 10q26, 11q12q13, 12p11p12, 12p13, 12q23q24, 14q31, 15q22q24, 16p11p13, 16q22q23, 17p11p13, 17q11q21, 17q22q24, 19p13, 19q13, 20q11q13, 21q22, 22q11q13)	MSS

6/02-329	serous	yes	invasive implants	46, XX[28]	no imbalances	no DNA available

7/02-828 A	Serous (right ovary)	yes	no	46, XX[13]/92, XXXX[7]	rev ish enh(4p15, 8q22q23, 13q22q31, 13q32), dim(1p32p36, 7p12p13, 7q11, 9q34, 11q12q13, 12p11p12, 12q23, 12q24, 15q22q24, 16p13, 17p12pter, 17q11q21, 19, 22q11q13)	MSS

8/02-829 B	serous (left ovary)	yes	no	46, XX[106]/92, XXXX[9]	no DNA available	--

9/03-325 A	serous (right ovary)	yes	no	46, XX[15]	rev ish enh(3p12p14, 3q13, 5p14, 6q15q16, 8q22q23, 9p21, 18q12), dim(1p31pter, 2q37, 7q11, 11q12q13, 12q24, 16p11p13, 17p11p13, 17q11q21, 17q23q25, 19p, 19q13, 22q11q13)	MSS

10/03-328 B	serous (left ovary)	yes	no	46, XX[15]	no imbalances	MSS

11/03-401	serous	no	no	Culture failure	rev ish enh(1p32pter, 1q21q22, 2p11p12, 2q37, 3p21, 4p16, 6p12p21, 9q33qter, 10q22q23, 10q24, 10q25, 10q26, 11q11q14, 12q24, 14q32, 15q22q25, 16p, 16q13qter, 17p, 17q11q22, 17q24qter, 19p13, 19q13, 20p11p12, 20q13, 22q11q13), dim(6q15q21, 6q22q24)	MSS

12/03-481	serous	yes	no	46, XX[32]	no imbalances	MSS

13/03-620 A	serous (right ovary)	yes	metastasis lympho node	46, XX, der(4) t(3;4) (q13;q34)[15]/46, XX[2]	no imbalances	MSS

14/03-621 B	serous (left ovary)	yes	metastasis lympho node	46, XX, der(4) t(3;4) (q13;q34)[10]/46, XX[5]	no imbalances	MSS

15/03-701	serous	no	no	46, XX[11]	no imbalances	MSS

16/04-36	serous	yes	invasive implants	49, XX, +3, +7, i(8)(q10), +12 [15]/50, idem, +r[2]/50, idem, -r, +mar[2]	rev ish enh(3, 7, 8q13qter, 12), dim(8p22pter)	MSS

17/04-682	mucinous	no	no	46, XX[3]	no DNA available	--

18/04-721	mucinous and serous	no	no	46, XX[18]	no imbalances	MSS

19/04-831 A	serous (left ovary)	yes	invasive implants	46, XX[84]	rev ish enh(2q24, 3p12, 3p13, 8q22q23, 13q22q31), dim(2q36q37, 7q35q36, 9q33q34, 10q25q26, 11q13, 12q23q24, 14q31q32, 15q22q24, 16p11p13, 17p11p13, 17q11q21, 17q22q25, 19p13, 19q13, 20q11q13, 22q)	MSS

20/04-832 B	serous (right ovary)	yes	invasive implants	47, XX, +7[18]	rev ish enh(Xq21q23, 2q22q32, 3p12p13, 3q12q13, 4q12q28, 5p13p14, 5q14q23, 6q12q21, 6q22, 7p12p21, 7q21q34, 8q13q21, 8q22q23, 9p21p24, 11q14q21, 13q21q31), dim(1p32pter, 2q37, 4p16, 6p23, 6q25q26, 9q34, 10q25q26, 11q12q13, 12q23q24, 14q31q32, 15q22q24, 16p11p13, 16q21q24, 17p, 17q11q21, 17q23q24, 19, 20q11q13, 21q22, 22q)	MSS

21/04-1211	mucinous	no	no	Culture failure	no imbalances	MSS

22/04-1213 A	serous (right ovary)	yes	invasive implants	46, XX[3]	rev ish enh(8p11p23, 8q11q24), dim(1p34p35, 15q11q13, 16p11p12)	MSS

23/04-1214 B	serous (left ovary)	yes	invasive implants	46, XX[3]	no DNA available	--

### Cell Culturing and Karyotyping

The tumor samples were manually minced and disaggregated with Collagen II (Worthington, Freehold, NJ, USA) until a suitable suspension of cells and cell clumps was obtained. After 6-7 days of culturing in a selective medium [18], colchicine was added and the cultures harvested according to Mandahl [19]. The chromosomes of the dividing cells were then G-banded and a karyotype established according to the recommendations of the ISCN [20].

### Fluorescence in Situ Hybridization (FISH) Analyses

BAC clones retrieved from the RPCI-11 Human BAC library and the CalTech human BAC library (P. de Jong libraries, http://bacpac.chori.org/home.htm) were used. The clones were selected according to their physical and genetic mapping data on chromosomes 3 and 6 as reported by the Human Genome Browser at the University of California, Santa Cruz website http://genome.ucsc.edu/. The clones specific for chromosome 3 were selected because they mapped to around the 3q13 breakpoint seen in three of the tumors we examined (see below and Table 2). The clones mapping on chromosome 6 spanned the region between markers D6S193 and D6S149, i.e., the consistent deletion reported by Tibiletti et al. [2] in the chromosomal region 167, 113, 548-167, 765, 926 in band 6q27 (Table 2). All clones were grown in selective media and DNA was extracted according to standard methods [21], DNA probes were directly labelled with a combination of fluorescein isothiocyanate (FITC)-12-deoxicytidine triphosphate (dCTP) and FITC-12-2-deoxyuridine triphosphate (dUTP), Texas Red-6-dCTP and Texas Red-dUTP (New England Nuclear, Boston, MA, USA), and Cy3-dCTP (GE Healthcare, UK) by nick translation. The subsequent hybridization conditions as well as the detection procedure were according to standard protocols [22]. The hybridizations were analyzed using a CytoVision system (Applied Imaging, Newcastle, UK).

**Table 2:** Clones Used for FISH Experiments.

BAC clone	Map position	UCSC position (hg18)
RP11-631J1	3q12.2	chr3:101, 560, 061-101, 723, 941

CTD-2303M9	3q13.2	chr3:113, 489, 978-113, 592, 063

RP11-514O12	6q27	chr6:167, 113, 548-167, 270, 484

CTD-2383F8	6q27	chr6:167, 253, 486-167, 374, 339

CTD-3184N3	6q27	chr6:167, 404, 540-167, 588, 046

RP11-931J21	6q27	chr6:167, 592, 153-167, 765, 915

RP11-178P20	6q27	chr6:167, 616, 370-167, 765, 926

### High-Resolution Comparative Genomic Hybridization (HR-CGH)

DNA was isolated by the phenol-chloroform method as previously described [23]. CGH was performed according to our modifications of standard procedures [24,25]. Chromosomes were karyotyped based on their inverted DAPI appearance and the relative hybridization signal intensity was determined along each chromosome. On average, 10-15 metaphases were analyzed. A negative (normal versus normal; the normal control was a pool of DNAs from four healthy women) and a positive (the colon cancer cell line LOVO with known copy number changes) control were included in the experiments. For the scoring of CGH results, we adopted the use of dynamic standard reference intervals (D-SRI). A D-SRI represents a "normal" ratio profile that takes into account the amount of variation detected in negative controls for each chromosome band. This provides a more objective and sensitive scoring criterion than fixed thresholds [26-28] and, consequently, a higher resolution. The D-SRI used was generated with data from 10 normal versus normal hybridizations (totalling 110 cells). This interval was automatically scaled onto each sample profile, and aberrations were scored whenever the case profile and the standard reference profile at 99% confidence intervals did not overlap. The description of the CGH copy number changes was based on the recommendation of the ISCN [20].

### Microsatellite Instability Status

Microsatellite instability (MSI) status was determined in all samples using a consensus panel of five microsatellite markers (BAT25, BAT26, D2S123, D5S346, and D17S250) [29]. A tumor was considered to be MSI-high if two or more of the five markers exhibited novel alleles compared to normal DNA, MSI-low if only one marker deviated from the normal pattern, and microsatellite stable (MSS) if none of the tumor genotypes showed an aberrant pattern. Control DNA corresponding to the individual tumors was not available from the patients and therefore single allele changes, i.e., the presence of two different alleles, can reflect a heterozygous constitutional genotype or a homozygous genotype with a novel tumor-specific allele. Thus, dinucleotide markers were not scored when such a pattern appeared in the tumors. The MSI status was assessed according to Wu et al. [30]. Allelic sizes were determined using GeneMapper 3.7 software (Applied Biosystems, Foster City, CA, USA) and the results were independently scored by two investigators. A second round of analyses was always performed and confirmed the findings.

## Results

The cell culturing and subsequent G-banding cytogenetic analysis gave informative results in 21 samples (Table 1), seven of which showed an abnormal karyotype whereas 14 were normal. The remaining two samples were culture failures and therefore could not be examined using this technique. All the cases with an abnormal karyotype had simple chromosomal aberrations. In three tumors, a single structural rearrangement was seen in a pseudodiploid karyotype: a t(3;17)(q13;q24) was detected in case 3 and a der(4)t(3;4)(q13;q34) was seen in cases 13 and 14, which were bilateral tumors from the same woman. In case 1, three unrelated clones with a single numerical aberration in each were identified. In case 16, three related clones were seen: 49, XX, +3, +7, i(8)(q10), +12[15]/50, idem, +r[2]/50, idem, -r, +mar[2]. Numerical changes only were found in three cases. Chromosomes 7 and 12 were most often involved in numerical changes (in three cases each, always as trisomies), whereas chromosomal band 3q13 was involved in the three cases showing only a structural rearrangement.

The HR-CGH gave informative results in 19 samples showing genomic imbalances in 11 of them (Table 1). From four lesions there was no DNA available for analysis. In six cases, the G-banding karyotype matched the pattern detected by CGH; five of them had a normal karyotype and showed no imbalances by HR-CGH whereas the last tumor (case 16) had numerical and structural changes all detected by both techniques. In six tumors, HR-CGH detected imbalances where G-banding analysis showed only normal karyotypes.

**Figure 2:**
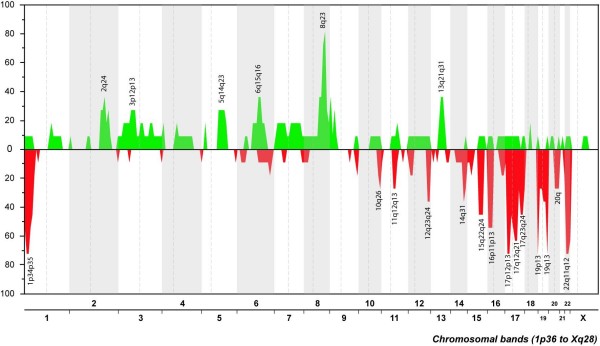
**The genomic imbalances detected by HR-CGH in 11 borderline ovarian tumors**. Gains are shown in green and losses in red color.

The tumors showed from five (samples 16 and 22) to 41 (sample 1) imbalances by HR-CGH with an average number of copy alterations (ANCA) index of 18.72. No amplifications were scored. The major copy number changes detected in the borderline tumors were gains from chromosome arms 2q, 6q, 8q, 9p, and 13q and losses from 1p, 12q, 14q, 15q, 16p, 17p, 17q, 19p, 19q, and 22q (Fig. 2). More specifically, the most frequently gained bands were, in order of decreasing frequency, 8q23 (82% of the cases showing imbalances), and 2q24, 6q15~16, 8q13~21, 9p23, and 13q22~31 (36%). The most frequently lost bands were 1p34~35, 17p12~13, 19p13, 19q13, and 22q11~12 (73%), 17q12~21 (64%), 16p11~13 (55%), 15q22~24, and 17q23~24 (45%), and 12q23~24 and 14q31 (36%).

The HR-CGH analysis gave informative results from both tumorous ovaries in two patients with bilateral disease (cases 13 and 14 and 19 and 20). The common imbalances found in these samples were gains of 2q24, 8q22~23, and 13q22~31 and losses of 9q34, 10q25~26, 12q23~24, 14q31~32, 15q22~24, 16p, 17p, 17q11~21, 17q23~24, 20q and 22q.

FISH was performed for two purposes: to characterize, possibly identify, the common breakpoint in 3q13 (seen in cases 3, 13, and 14; the latter two were from bilateral tumors in the same woman) and to test for the consistent deletion previously found in borderline ovarian tumors by Tibiletti et al. [2]. For the former purpose, FISH was performed on cases 13 and 14 on previously hybridized (stripped) slides; however, we did not get informative results. To examine for 6q deletions, FISH was performed on a total of 12 tumors. In nine cases, newly dropped slides were made, whereas in three cases old slides previously used for other FISH experiments were stripped and used. Because no metaphase spreads were available for FISH analysis, interphase nuclei were used to check for the reported deletion on 6q. A total of 200 nuclei per sample were analyzed but no indication of a deletion of the alleged 6q target region was detected in the nine cases yielding informative results.

The testing for MSI gave informative results in 18 tumors. All of them were classified as microsatellite stable (MSS) as none of the tumor genotypes showed an aberrant pattern. The remaining five samples were not analyzed because there was no DNA available.

## Discussion

FISH experiments were performed to investigate whether the about 300 kb deletion in 6q27 found so consistently by Tibiletti et al. [2] in borderline ovarian tumors was a feature also of the tumors of our series. In none of nine informative cases (five with a normal karyotype, four with clonal chromosome abnormalities) did we see any such deletion. We cannot offer any biological explanation for the discrepant results, and so future studies will be necessary to find out what is more typical of borderline tumors.

MS status has previously been analyzed in a total of 112 ovarian tumors of borderline malignancy, 14 of which showed instability for one or more of the markers used. However, some studies were performed before the consensus reached by NCI for evaluating MSI [29] and therefore differences in the type and number of microsatellites can be found in these studies [31-36]. All 18 informative borderline ovarian tumors examined by us turned out to be microsatellite stable (MSS). Based on the results of our and the latest other studies [34-36], it therefore seems that at least the great majority of ovarian tumors of borderline malignancy tend to have a stable MS pattern.

The pattern of chromosomal aberrations seen by G-banding analysis in the present study with gains of chromosomes 7 and 12 as recurrent changes is largely similar to that previously found in abnormal karyotypes of ovarian borderline tumors and well differentiated carcinomas [8,9]. Poorly differentiated and/or advanced stage ovarian carcinomas, on the other hand, tend to have more complex karyotypes with multiple numerical as well as structural aberrations [18,37]. A novel finding, however, was that three tumors (cases 3, 13, and 14; admittedly, the last two were from the same patient) showed a single structural aberration that seemed to involve chromosomal band 3q13. Unfortunately, we did not have left fixed cells in suspension to perform FISH experiments on newly dropped slides, and our attempts to use stripped slides for better FISH characterization failed. Nevertheless, the detected G-banding similarity hints that one or more genes mapping to this band may play a pathogenetic role in a subset of borderline ovarian tumors.

Most tumor karyotypes in the present series were normal, as only seven of 21 successfully cultured samples showed clonal chromosome abnormalities. The simplest explanation for this is that the cells carrying aberrations did not divide *in vitro *and therefore could not be detected by G-banding analysis. Confirmation that this was indeed so stems from the observation that six tumors with a normal karyotype showed genomic imbalances by HR-CGH. However, in the five tumors where both G-banding and HR-CGH analyses gave a normal karyotype and no imbalances, one must assume that either no aberrations were present in at least a substantial minority of the cells or they were too small to be seen at the chromosomal resolution level.

The major copy number changes detected in the borderline tumors were gains of chromosomal bands or regions 8q23 (present in 82% of the cases showing imbalances), 2q24, 6q15~16, 8q13~21, 9p23, and 13q22~31 (36%), and losses of 1p34~35, 17p12~13, 19p13, 19q13, 22q11~12 (73%), 17q12~21 (64%), 16p11~13 (55%), 15q22~24, and 17q23~q24 (45%), and 12q23~24 and 14q31 (36%). Some of these imbalances have already been reported by other groups such as gain of 8q and losses of 1p and chromosome 17 [12,14-16,38]. However, the use of HR-CGH allowed us to increase the resolution and narrow down the mentioned regions to 8q23, 1p34~35, 17p12~13, 17q12~21, and 17q23~24. Additional studies are needed to better investigate the nature of the gene(s) present here that may be involved in the genesis or progression of ovarian borderline tumors.

Much interest has focused on the loss of genetic information from chromosome 17 in ovarian tumors. In the short arm, losses seem to occur especially at 17p13.3 [39-41] with *OVCA1 *and *OVCA2 *as possible target tumor suppressor genes [42]. However, proximal 17p changes have received more attention. Mutation of the gene *TP53 *in 17p13.1 is the most common genetic alteration thus far detected in ovarian cancer, with mutation rates as high as 50% in advanced stage carcinomas [43]. The frequency of *TP53 *alterations varies depending on whether the tumors are benign, borderline, or malignant as well as on the histological subtype, i.e., serous, mucinous, endometrioid, and clear cell ovarian carcinoma. In benign epithelial ovarian tumors no mutation of *TP53 *has been described [44,45]. In borderline tumors, *TP53 *mutation and over-expression may occur, but are not common [46-48]. In malignant tumors, the prevalence of *TP53 *gene mutations increases with increasing stage [44]. In the long arm of chromosome 17, losses at 17q12~21 are frequently observed in ovarian carcinomas [39,49], but this is the first time that chromosomal regions 17q12~21 and 17q23~24 are identified as lost in ovarian borderline tumors. The breast and ovarian cancer susceptibility gene *BRCA1 *maps to 17q21 and could be one possible gene target, but the actual pathogenetic involvement of this and other genes located in 17q needs to be further investigated.

The present series of borderline ovarian tumors is the largest one hitherto analyzed for genomic imbalances and the first examined by HR-CGH. In addition to the above-mentioned imbalances, we also identified some new chromosomal regions gained at a high frequencies, i.e., 2q24, 6q15~16, 8q13~21, 9p23, and 13q22~31 (36%), as well as losses of 19p13, 19q13, and 22q11~12 (73%), 16p11~13 (55%), 15q22~24 (45%), and 12q23~24 and 14q31 (36%). Again, further studies are needed to investigate the possible involvement of genes present here in ovarian tumorigenesis. The aberrations found in the two histological subtypes of borderline tumors (serous versus mucinous) were also compared but no specific difference was noted.

The present series included five patients with bilateral borderline tumors. Informative results were obtained by HR-CGH from both tumorous ovaries in two patients (pairs 13 and 14 and 19 and 20). Cases 13 and 14 showed the same unbalanced 3;4-translocation by karyotyping in both tumorous ovaries. This is a sure sign that the bilateral tumors were part of a single neoplastic process and, hence, that one of them must have occurred by a metastatic mechanism. No imbalances were seen by HR-CGH in this tumor pair, probably because too little was contributed by cells of the neoplastic parenchyma to the total DNA extracted. In cases 19 and 20, a +7 was seen in one tumor whereas the other showed a normal karyotype; this technique therefore did not yield certain information as to the two tumors' clonal relationship. However, common imbalances were found by HR-CGH such as gains of 2q24, 8q22~23, and 13q22~31 and losses of 9q34, 10q25~26, 12q23~24, 14q31~32, 15q22~24, 16p, 17p, 17q11~21, 17q23~24, 20q and 22q. The data are too small for anything but speculations, but it is possible that these bands/regions may carry gene(s) important for the development of bilateral borderline ovarian tumors. It is in this context intriguing that the same imbalances also occurred in some of the other bilateral tumors, albeit then found in only one tumorous ovary while the other was uninformative. But regardless of what, if any, pathogenetic changes might contribute to the development of bilateral borderline tumors particularly, the combined karyotypic/CGH data on the two only completely informative pairs strongly indicate that bilaterality occurs by spreading from one side to the other, not as two clonally separate processes.

The average number of copy alterations per tumor calculated in the present series was 18.72. It is interesting to note that for the bilateral borderline ovarian tumors the ANCA index was 24.5 whereas for the unilateral borderline ovarian tumors it was 17.44. This difference, small though it may seem, is consistent with the interpretation that bilateral tumors reflect a more advanced disease stage compared with unilateral ones, inasmuch as they arise via the spreading process referred to above.

## Conclusion

The introductory question as to whether borderline tumors of the ovary represent a transitional stage from benign to clearly malignant or a pathogenetically "closed" tumor type of its own, without a tendency to further progression, remains, perhaps not surprisingly, unanswered by the findings of the present study. It may be worthy of note, however, that two main genomic groups of tumors were discerned in this series, one (n = 5) showing a normal karyotype and no imbalances detectable by HR-CGH and the other (n = 14) showing aberrations by one or both analytical methods. Possibly, and we underscore that this is presently only a speculation, tumors of the first group are more developmentally stable and may have no propensity to progress to more malignant carcinomas, whereas those of the second group with chromosomal/genomic aberrations may undergo further evolutionary changes giving rise to a more malignant phenotype. The fact that gain of chromosomal band 8q23, as well as losses of 19p13 and 19q13, feature prominently in both overt carcinomas [37,50] and in the present series (the gains were found in 5 of 5 cases with bilateral borderline tumors and in 4 of 6 informative unilateral tumors showing imbalances) fits, but by no means proves, this hypothesis. To further validate it would require more extensive studies that should not only compare the karyotypic/genomic findings of borderline and malignant tumors, but should also collate these findings with clinical information on the same group of patients.

## Competing interests

The authors declare that they have no competing interests.

## Authors' contributions

FM conducted the study, participated in design, coordination, data interpretation, and drafted the manuscript. LH participated in karyotyping and FISH experiments. TA and RAL participated in MS status analysis and discussion of data. HKA participated in FISH analysis. VMA and BD performed the pathological diagnosis of each tumor and provided samples for cytogenetic analysis. CGT provided samples and clinical information. SH participated in the design and coordination of the study and critically revised the manuscript. All authors read and approved the final manuscript.
